# She called that thing a mido, but should you call it a mido too? Linguistic experience influences infants’ expectations of conventionality

**DOI:** 10.3389/fpsyg.2015.00332

**Published:** 2015-03-27

**Authors:** Annette M. E. Henderson, Jessica C. Scott

**Affiliations:** Early Learning Laboratory, School of Psychology, The University of Auckland, Auckland, New Zealand

**Keywords:** conventionality, bilingualism, experience, infant cognition, visual habituation, infants

## Abstract

Words are powerful communicative tools because of conventionality—their meanings are shared among same language users. Although evidence demonstrates that an understanding of conventionality is present early in life, this work has focused on infants being raised in English-speaking monolingual environments. As such, little is known about the role that experience in multilingual environments plays in the development of an understanding of conventionality. We addressed this gap with 13-month-old infants regularly exposed to more than one language. Infants were familiarized to two speakers who either spoke the same (English), or different (French vs. English) languages. Next, infants were habituated to a video in which one of the speakers provided a new word and selected one of two unfamiliar objects. Infants were then shown test events in which the other speaker provided the same label and selected either the same object or a different object. Our results demonstrate that exposure to at least one other language influences infants’ expectations about conventionality. Unlike monolinguals, bilingual infants do not assume that word meanings are shared across speakers who use the same language. Interestingly, when shown speakers who use different languages, bilingual infants looked longer toward the test trials in which the second speaker labeled the object consistently with the first speaker. This finding suggests that exposure to multiple languages enhances infants’ understanding that speakers who use different languages should not use the same word for the same object. This is the first known evidence that experience in multilingual environments influences infants’ expectations surrounding the shared nature of word meanings. An increased sensitivity to the constraints of conventionality represents a fairly sophisticated understanding of language as a conventional system and may shape bilingual infants’ language development in a number of important ways.

## Introduction

Words are powerful communicative tools because their meanings are shared within a particular linguistic community (e.g., Clark, 1992, 1993, 2009). A basic understanding of this fact about language emerges early in development (Henderson and Graham, 2005; Graham et al., 2006; Buresh and Woodward, 2007; Henderson and Woodward, 2012) and has been argued to play an important role in children’s word learning (for reviews see Sabbagh and Henderson, 2007, 2013; Diesendruck and Markson, 2011). Although the existing evidence clearly suggests that an understanding of conventionality emerges early in life, the focus of these studies has been on infants being raised primarily in monolingual English-speaking environments. As such, very little is known about the role that experience in multilingual environments plays in the development of an understanding of conventionality. The present research begins to fill this gap by providing the first known investigation of the role that exposure to multilingual environments plays in infants’ expectations surrounding the shared nature of words.

The conventional nature of language refers to the fact that “For certain meanings there is a form that speakers expect to be used in a language community” (Clark, 1992, p. 171). This shared nature of words ensures consistency in word meanings across language-users and thus, regulates communication within linguistic communities. Critically however, conventionality also has constraints; linguistic conventions are bounded by linguistic groups (Clark, 2007). To illustrate, although all English speakers would be expected to share knowledge that the word “shoes” refers to a category of items that are put on one’s feet for protection, users of other languages are not bound by the same expectation. For example, French speakers would instead be expected to say “les chaussures” to refer to footwear. Thus, a key challenge facing language learners is to acquire the word meanings that are known and used within their linguistic community.

There is now converging evidence from a number of studies suggesting that children are sensitive to various aspects relevant to the conventional nature of language early in their lives. By 12 months of age, infants are aware that speech is the tool that people use to communicate (Martin et al., 2012; Vouloumanos et al., 2014; Pitts et al., 2015). By 16 months, infants are sensitive to the fact that objects have conventional names and expect other people to use the conventionally appropriate names for objects (i.e., call a “ball” a ball and not “a shoe”; Koenig and Echols, 2003). By their second birthday, infants focus on learning words, and not other symbolic behaviors, such as sounds or gestures, as appropriate names for objects (Namy and Waxman, 1998; Woodward and Hoyne, 1999; Graham and Kilbreath, 2007).

The most direct evidence of an understanding of the conventional nature of words comes from studies testing the age at which children understand that object labels are shared across people who use the same language (Woodward et al., 1994; Henderson and Graham, 2005; Graham et al., 2006; Buresh and Woodward, 2007; Henderson and Woodward, 2012). To test this question in infants, Buresh and Woodward (2007) developed a visual habituation paradigm in which infants were repeatedly shown an event in which a speaker either provided a novel label (i.e., “medo”) or expressed positive affect (i.e., “ooh. Mmmmm.”) while holding one of two novel objects. After habituating to that event, infants were shown test trials in which a speaker produced the same label while holding either the previously labeled target object (target trials) or a different object (distractor trials). The key manipulation was whether the test speaker was the same speaker from habituation, or a different speaker who had been shown to use the same language as the habituation speaker. Buresh and Woodward’s results revealed that, regardless of test speaker, 12-month-old infants looked longer toward the distractor test trials in the word conditions but not the positive affect condition (see Henderson and Woodward, 2012 for similar results with 9-month-olds). Thus, by 9 months, infants demonstrate an understanding of the shared nature of word meanings—they expect object labels, but not object preferences to be generalized across speakers.

However, word meanings are also tied to specific linguistic communities. Thus, a sophisticated understanding of conventionality requires understanding of the scope of its application; that word meanings are only shared by individuals from *the same* linguistic community. Au and Glusman (1990) demonstrated that preschool-aged children show some understanding of this concept in a study in which monolingual English speaking children are taught a novel label for an animal in two languages (i.e., English and Spanish). The children were then asked a question (“Can you guess which one Spanish speaking children would call a theri?”). The results showed that 3- to 6-year-olds would accept two labels for the same object, but only if the evidence was clear that the labels came from different languages. This finding suggests that preschool-aged children understand that labels are constrained by linguistic community insofar as speakers of different languages use different labels.

Scott and Henderson (2013) provided the first evidence of an understanding of the fact that linguistic community constrains conventionality in monolingual infants. In this study, 13-month-olds being raised in monolingual-English environments were familiarized to two speakers singing nursery rhymes in different languages (one actor sang in French and the other in English). Infants were then habituated to one of the speakers providing a new word (i.e., “A modi. A modi.”) while holding one of two unfamiliar objects. After habituation, the second speaker provided the test events in which he/she uttered the same word as in habituation and picked up either the same or different object. Contrary to the results of the different speaker conditions reported in past research in which the speakers had been shown to use the same language (i.e., Buresh and Woodward, 2007; Henderson and Woodward, 2012), infants in Scott and Henderson’s study did not look longer toward either test event. That is, infants did not generalize the word-referent link across two speakers who had been shown to speak different languages. These findings suggest that infants as young as 13 months of age have a fairly nuanced understanding of conventionality; they are sensitive to the fact that linguistic community constrains conventionality.

Taken together, the existing evidence provides a clear picture that infants understand several facets of the conventional nature of language. However, because this work has focused on infants being raised in monolingual environments, very little is known about the extent to which experience in multilingual environments influences the development of an understanding of conventionality. Given that as many as half the world’s children grow up exposed to more than one language (Hoff, 2009), investigating the role that multilinguistic experience plays in the early development of an understanding of conventionality represents a significant gap in the literature.

Similar to infants who are exposed to one language, bilingual infants are exposed to people (within a linguistic community) labeling objects consistently. However, unlike monolingual infants, bilingual infants also receive direct evidence that an object can have more than one label. For instance, while a bilingual infant’s English-speaking father will always use the word “shoes” when placing shoes on his infant’s feet, the infant’s French-speaking mom will always say “les chaussures” while doing so. In addition to receiving direct evidence that objects can have two names, bilingual language learners are likely to have firsthand experience with the fact that linguistic community constrains conventionality. Infants may have already tried to use a word in one of their languages with a speaker from an entirely different linguistic community and thus, encountered a situation in which a word they know is not shared by another person. Such experiences might encourage bilingual infants to develop an early appreciation of the fact that not all people are likely to share many of the words they might be learning. Given that bilingual infants are provided with regular exposure to multiple labels for an object and may be more likely to have had experiences in which other language users do not understand the words that they use, it seems reasonable to expect that they may develop different expectations surrounding conventionality.

Experience with early bilingualism has been shown to influence young children’s expectations about language in a number of ways. For example, early bilingualism has been shown to result in an increased awareness of the arbitrary nature of language. Evidence supporting this point comes from Eviatar and Ibrahim (2000) who explained an exchanging words game (e.g., “We’ll call the sun the moon and the moon the sun”) and then asked the children to answer a question (e.g., “When you go to sleep at night what do you see in the sky?”). The results of this study revealed that 4- to 7-year-old bilingual children were more likely to adhere to this new relationship than were monolingual children on this task suggesting a greater appreciation of the arbitrary nature of word-referent links. Similarly Bialystok (1988) showed that bilingual children perform better than their monolingual counterparts in tasks of metalinguistic awareness, or knowledge about language. However, only a handful of studies have examined whether early exposure to more than one language influences children’s expectations about conventionality.

One such study was conducted by Diesendruck (2005) in which monolingual and bilingual 3-year-old children were taught a new word for one of two objects (e.g., “This is a Teega”). In a subsequent task, children were asked to select the object that was the referent of a second novel label (e.g., “Can you give me the patoo?”) by a speaker who was absent when the original word was taught. Consistent with past research conducted by Diesendruck and Markson (2001), monolingual 3-year-olds assumed conventionality; they assumed that the second speaker was aware of the previously labeled object’s name and when a different term was used, they inferred that the second novel label was used to refer to the unlabeled object. Interestingly, bilingual children did not select the unlabeled object at levels greater than chance. These findings suggest that bilingual children do not assume that a speaker who was absent when an object had been labeled will know (and use) the same word to refer to the same object and thus, did not assume that the second novel label would refer to the unlabeled object. These results suggest that that bilingual preschoolers are cautious about making assumptions that other people will share knowledge of the linguistic terms that they know. Diesendruck concluded that bilingual children might believe that there are conventional ways to refer to objects, but do not assume that everybody knows them. Consistent with this possibility are the recent findings bilingual preschool-aged children (Kalashnikova et al., 2014) and toddlers (Byers-Heinlein et al., 2014) will accept a second label for a novel object from a second speaker if that speaker has been shown to use a different language. Together, these findings suggest that bilingual children do not assume conventionality and are sensitive to the fact that object labels do not generalize across linguistic groups.

Taken together, existing evidence suggests that early exposure to both consistent labeling within a linguistic community and divergent labeling from different language speakers influences bilingual children’s assumptions about the use of labels. As noted above, bilingual children are cautious in making assumptions that speakers share linguistic terms (Diesendruck, 2005) and understand that object labels are not shared across speakers of different languages (Byers-Heinlein et al., 2014; Kalashnikova et al., 2014). Considering these findings and in light of increasing evidence of an understanding of conventionality in infancy, it is possible that bilingual infants may show similar tendencies. Some reason to suspect that bilingual infants might be attuned to the role that linguistic community plays in conventionality comes from evidence suggesting that bilingual infants are able to distinguish between different languages early in their lives. There is now a solid body of evidence demonstrating that, early in infancy, there are perceptual discrimination abilities, which assist infants in differentiating between languages (e.g., Bahrick and Pickens, 1988; Mehler et al., 1988; Moon et al., 1993; Bosch and Sebastián-Gallés, 1997). Some researchers have argued that these discriminative abilities allow bilingual infants to form separate representations for the languages they are acquiring (for a review see Werker and Byers-Heinlein, 2008). Regularly making this kind of distinction when receiving language input may result in bilingual infants being particularly sensitive to the presence of different languages. Supporting this sensitivity is evidence suggesting that bilingual toddlers adjust their language use based on the language most relevant to the present context, even when their communicative partner is an unfamiliar adult, which suggests a well-developed understanding of how and when to use their different languages (e.g., Genesee et al., 1996; Deuchar and Quay, 1999). These findings indicate that from early on, bilingual language learners show an awareness of the linguistic community of the speakers around them and raise the possibility that infants might be particularly sensitive to the fact that speakers of different languages should not use the same word meanings.

We investigate whether experience in a bilingual environment influences infants’ understanding of conventionality in the present research by using a visual habituation paradigm. Thirteen-month-old infants who are being raised in bilingual environments were familiarized to two speakers singing nursery rhymes in one of two conditions. Infants were exposed to two speakers singing nursery rhymes either in the same language (i.e., both speakers sang in English) or in a different language (i.e., one speaker sang in English and the other in French). Infants were then habituated to one of the speakers providing a novel label (i.e., “medo”) while holding one of two novel objects. After habituation, infants were shown test trials in which the other speaker (from familiarization) produced the same label while holding either the previously labeled object (target trials) or a different object (distractor trials). If infants being raised in bilingual environments have the same expectations of conventionality as infants being raised in monolingual environments, we expected our findings to be consistent with previous research. Specifically, we expected that: (1) infants in the same language condition would look significantly longer toward the distractor test trials thereby demonstrating an expectation that word-referent links are shared across speakers who have been shown to use the same language and (2) infants in the different language condition would not look significantly longer toward the distractor test events thereby demonstrating an understanding that word-referent links are not shared by speakers who do not use the same language. To our knowledge, this is the first investigation of an understanding of the constraints of conventionality in bilingual children under the age of 3.

## Materials and Methods

### Participants

Thirty 13-month-old infants (*M*_age_ = 13 months, 5 days; SD = 0.39; range = 12;2–13;29; 16 males) being raised in multilingual environments were recruited from a large database of families who have volunteered to take part in studies on infant development managed by a cognitive development lab in an urban center in New Zealand. Parents reported their infant as being exposed to English between 40 and 65% of the time (*M* = 53.57%, SD = 7.6%). Thus, infants were exposed to at least one other language a minimum of 35% and a maximum of 65% of the time. The other languages to which infants were exposed were: German (*n* = 5), Dutch (*n* = 2), Samoan (*n* = 2), Portuguese (*n* = 3), Chinese (*n* = 3), Maori (*n* = 2), French (*n* = 2), Farsi (*n* = 2), Serbian (*n* = 1), Italian, (*n* = 1), Korean (*n* = 1), Turkish (*n* = 1), Spanish (*n* = 1), Afrikaans (*n* = 1), Hindi (*n* = 1), Polish (*n* = 1), and Japanese (*n* = 1)^1^. Parents also reported the ethnicities of their infant, which resulted in the following breakdown: New Zealand European (*n* = 6), Pacific Islander (*n* = 1), Asian (*n* = 3), Middle Eastern (*n* = 1), and other European (*n* = 5). Thirteen infants were reported as belonging to more than one ethnic group. One parent did not complete the demographic questionnaire.

Infants were randomly assigned to either the ***same language condition*** (*n* = 14, 8 males, 6 females) or the ***different language condition*** (*n* = 16, 8 males, 8 females). An additional seven infants participated but were excluded from the final sample due to technical errors (*n* = 3) or because the infant received more than 65% of English exposure and thus did not meet the bilingual language criteria (*n* = 4).

Infants were given a small prize for their participation at the end of the study; parents were given a parking ticket and a $10 gift voucher for petrol or groceries.

### Materials, Stimuli, and Procedure

After a warm-up play period during which infants were given time to become comfortable in the laboratory environment and the experimenter completed the informed consent procedures with the parent, infants and their parents were escorted to the experimental testing room.

Infants were seated on their parents lap approximately 168 cm from a projector screen on which the video stimuli would be shown. The presentation of the video stimuli was controlled by the experimenter who stood behind a curtain via a MacBook Pro laptop. The software Looking Time X (Hannigan, 2008) was used to present the video stimuli. Infants’ gaze was recorded using a camera that was hidden underneath the projection screen, which was connected to a mixer that consolidated the video stimuli with the view of the infant from the Baby Camera into one video file. This video file was recorded using a HyperDeck Studio SSD recording device. All of the recording equipment was hidden behind a curtain out of infants’ view. The live feed from the baby view camera was also transmitted via HDMI to a monitor in an adjacent room in which the coder, who was blind to condition and trial, sat and coded infants’ attention.

Once the infant was seated on his/her parent’s lap, the experimental session began. All infants participated in the following six phases (as per Scott and Henderson, 2013): language familiarization, habituation, baseline, test familiarization, and test.

#### Language Familiarization

**FIGURE 1:**
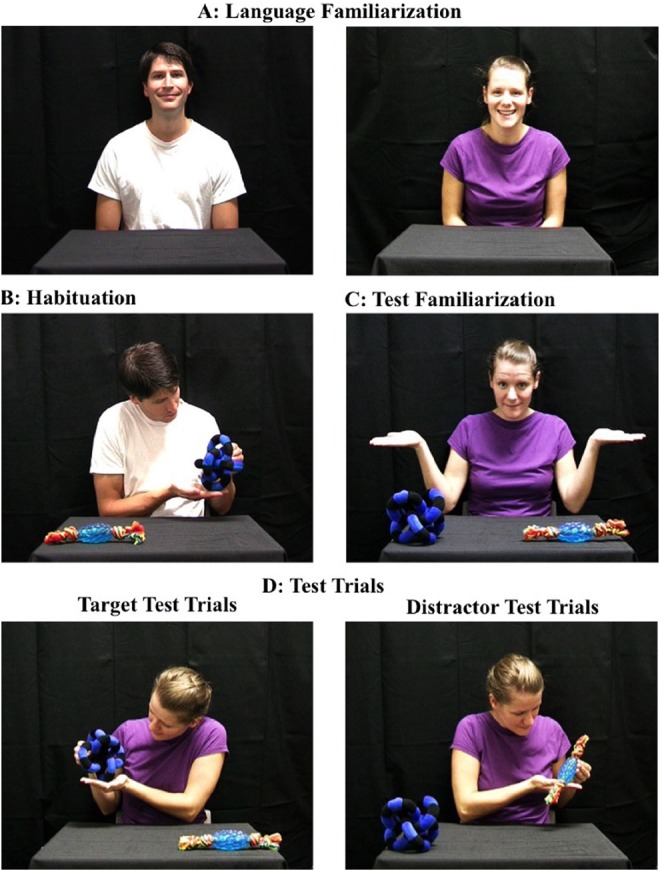
**Sample image of the language familiarization, habituation, test familiarization, and test trials used in this research**.

During this 90-s phase infants were introduced to two speakers, a male and a female, who alternated singing nursery rhymes (see Figure 1). For infants in the ***same language condition***, both speakers sang in English; the male speaker sang “Mary Had a Little Lamb” and “Itsy Bitsy Spider,” the female speaker sang “Row, Row, Row Your Boat” and “Twinkle Twinkle Little Star.” Consistent with Scott and Henderson (2013), infants in the ***different language condition*** were shown the male speaker singing in French (e.g., “Frere Jacques” and “Alouette”) and the female speaker singing in English (e.g., “Row, Row, Row Your Boat” and “Twinkle Twinkle Little Star”). The female actor was a native English speaker. The male actor was both a native French and English speaker and thus, did not have a French-accent when singing the English nursery rhymes. Although each song differed slightly in duration, the total duration of time infants were exposed to each speaker was consistent within and across conditions. To ensure that there were no differences across conditions in infants’ attention during this phase we ran a 2 (song: first, second) × 2 (speaker: male, female) × 2 (condition: same language, different language) mixed-design ANOVA on the percentage of time that infants looked toward the display for each song with song and speaker as within subject factors. This analysis revealed a significant main effect of speaker, *F*(1,23) = 19.37, *p* < 0.001, η^2^ = 0.46; infants spent a significantly smaller percentage of time attending to the display during the male speaker’s songs (*M* = 92.4%, SD = 1.52) than they did the female speaker’s songs (*M* = 99.1%, SD = 0.49). Importantly, no other effects reached statistical significance confirming that there were no differences between conditions in the percentage of time that infants attended to either the songs and/or the speakers during this phase. An independent samples *t*-test further confirmed that the duration of time (seconds) that infants spent looking toward the speakers during this phase was not significantly different across conditions (*M*_same language_ = 77.0 = SE_same language_ = 2.04; *M*_different language_ = 73.33, SE_different language_ = 1.02), *t*(25) = 1.75, *p* > 0.05, Cohen’s *d* = 0.66.

#### Habituation

Infants in both conditions were shown the same habituation event in which the male speaker looked up from his lap, smiled, looked at one of the two objects on the table, provided a new word (i.e., “medo”), picked up the object, and said “medo” a second time while looking at the object in his hand (see Figure 1). Infants were shown this video until the sum of their attention toward three consecutive trials was less than the sum of the first three habituation trials divided by two. (i.e., the habituation criterion), or until 14 habituation trials had elapsed.

#### Baseline

After habituation, infants were shown the habituation event one last time before entering the next phase.

#### Test Familiarization

The purpose of this trial was to introduce infants to the set-up for the test trials. The second speaker (from the language familiarization phase) was seated at the table between the two objects from habituation. Consistent with other habituation studies (e.g., Woodward, 1998; Buresh and Woodward, 2007; Henderson and Woodward, 2012; Scott and Henderson, 2013), the side on which each object appeared was switched. During this trial, the speaker looked up smiled, looked at each object and then back toward the infant, lifted her arms up and shrugged (i.e., as if to say “which one?”). The non-verbal nature of this trial ensured that infants were not reminded of the language used by the second speaker ensuring that any condition differences were a result of the language information provided to infants during the language familiarization phase.

#### Test Trials

Infants in both conditions were shown the same test trials in which the second speaker provided the word “medo” and grasped either the same object that the first speaker had grasped during habituation (i.e., target test trials) or the other object that was present during habituation but never grasped (i.e., distractor test trials). All infants were shown three test trials of each type in alternation.

After the last test trial, infants and their parents were escorted back to the family room. Parents were told about the hypotheses of the study and were given the opportunity to ask any questions. After answering their questions, the experimenter thanked parents for their time, gave the infant his/her prize and parents their voucher and parking ticket and then walked the family back to the carpark.

All phases of the experiment, with the exception of language familiarization, were infant controlled. Thus, the video paused after the actor had provided the second label and/or stopped moving and remained on the screen until the infant looked away for 2 s, or until 120 s had elapsed. The paused frame marked the onset of the calculation of infants’ looking time. Infants’ looking time and habituation criterion was calculated using the software jHab (Casstevens, 2007). The target object and type of first test trial were counterbalanced across conditions.

A second coder reliability coded all of the habituation and test trials. The original coder and the second coder agreed on 93% of the test trials. Importantly, the direction of disagreements was not systematic across the types of test trials (Fisher’s Exact Test, *p* = 0.56, two tailed).

## Results

Preliminary independent samples *t*-tests revealed no significant differences between conditions in infants’ age [*t*(28) < 1], percentage of time exposed to English [*t*(28) = 1.34, *p* > 0.1], or number of languages to which infants were exposed [*t*(28) = 1.74, *p* = 0.08]. Next, we investigated whether infants’ attention during the habituation phase differed depending on the condition to which infants were assigned. Table 1 shows infants’ average looking time during habituation and toward the test familiarization trial. As expected, a 2 (habituation trial: sum first three trials, sum last three trials) × 2 (condition: same language, different language) mixed-design ANOVA revealed that infants looked significantly longer on the first three habituation trials (*M* = 52.49, SE = 4.96) than they did on the last three habituation trials (*M* = 19.34, SE = 1.67), *F*(1,28) = 66.09, *p* < 0.001, η^2^ = 0.70. Critically, there were no differences between the conditions in infants’ attention during the habituation phase.

**TABLE 1:** **Mean looking times and standard errors for the habituation, baseline, and familiarization phases for each condition**.

	Habituation			Test familiarization
	Sum first 3	Sum last 3	Baseline	
Condition				
Same language	49.90(4.91)	18.54(1.97)	7.21(1.50)	21.34(2.33)
Different language	55.08(8.14)	20.15(2.61)	13.32(2.00)	23.71(6.53)

Independent samples *t*-tests revealed no significant differences between conditions in the average number of habituation trials or the average duration of the test familiarization phase, *t’s*(28) < 1. Surprisingly, infants in the different language condition (*M* = 13.33, SE = 2.00) looked significantly longer toward the baseline trial than did the infants in the same language condition (*M* = 7.21, SE = 1.51), *t*(28) = 2.38, *p* = 0.02, Cohen’s *d* = 0.87. In light of this finding, the main analyses were conducted controlling for infants’ attention toward the baseline condition.

**FIGURE 2:**
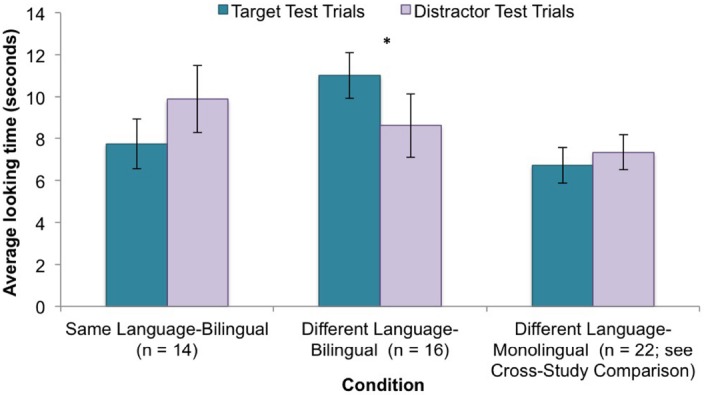
Infants’ average looking time ( ± SE) toward each type of test trial for each condition * = *p* < 0.05.

Of key interest was whether infants’ attention toward the target and distractor test trials differed depending on whether the speaker conducting the test trials had previously been shown to speak a language that was either the same as, or different from, the speaker who conducted the habituation trials. This question was examined by running a 2 (test trial type: target, distractor) × 2 (condition: same language, different language) × 2 (first test trial: target, distractor) mixed-design ANCOVA on infants’ attention toward the test trials with test trial type as the within-subjects factor and attention toward baseline as the covariate^2^. The results revealed a statistically significant interaction between test trial type and condition, *F*(1,25) = 4.31, *p* < 0.05, η^2^ = 0.15 and no other significant effects (see Figure 2).

A follow-up independent samples *t*-test revealed that infants in the same language condition did not look significantly longer toward either type of test trial, *t*(13) = 1.37, *p* > 0.1, suggesting that infants did not expect two speakers who had previously been shown to use the same language to use the same word-object pairings. Thus, the infants in this study did not generalize the word-referent link across speakers from the same linguistic community. In contrast, infants in the different language condition looked significantly longer toward the target test trials than they did the distractor test trials, *t*(15) = 2.20, *p* < 0.05, Cohen’s *d* = 0.60, *r* = 0.63, suggesting that infants found it unexpected when two speakers who had previously been shown to use different languages used the same word to refer to the same object, but not when the speakers used the same word to refer to a different object.

A sign test revealed that 79% of infants in the same language condition looked longer toward the *distractor* trials than they did the target test trials, *p* = 0.057. The fact that the *p*-value for this analysis is approaching significance contrasts with the results of the ANCOVA results reported above, which suggests that the infants in this study may have some, but not a robust, expectation that word-referent links are to be used consistently across two speakers who have been shown to use the same language. Conversely, 81% of the infants in the different language condition looked longer toward the *target* test trials than they did toward the distractor test trials (*p* = 0.02, sign test). This finding further suggests that bilingual infants do not generalize word-referent links across speakers who have been shown to speak different languages and, in fact, they find it surprising when word-referent links are used consistently across two speakers that had been shown to use different languages.

In sum, the results of the same language condition show that infants being raised in bilingual environments do not have a robust expectation that word-referent links are shared across speakers who have been shown to speak the same language. The results of the different language condition demonstrate that 13-month-old infants being raised in bilingual environments do not expect two speakers who had previously been shown to speak different languages to use the same word to refer to the same object.

### Comparison of Infants being Raised in Multilingual vs. Monolingual Contexts: Different Language Condition

To further examine the role that linguistic experience plays in infants’ expectations surrounding the constraints of conventionality, we conducted a final set of analyses comparing the looking time data of the bilingual infants in this study to a group of monolingual infants (*n* = 22; 12 males, 10 females; mean age = 12 months, 27 days; range = 12 months, 9 days to 13 months, 21 days). Eighteen of the monolingual infants were from the data reported in Scott and Henderson (2013, Experiment 1)^3^ and the remaining four were the infants who had been excluded from the final sample of this study because they did not meet the language criteria (i.e., infant had more than 65% of English exposure). As expected, the bilingual infants were exposed to significantly less English (*M* = 51%, SE = 2.19) than were the monolingual infants (*M* = 94%, SE = 2.34), *t*(36) = 12.98, *p* < 0.001, Cohen’s *d* = 4.38. The bilingual infants were also exposed to a significantly greater number of languages (*M* = 2.19, SE = 0.10) than were the monolingual infants (*M* = 1.55, SE = 0.14), *t*(36) = 3.40, *p* < 0.002, Cohen’s *d* = 1.15.

Preliminary analyses did not reveal any statistically significant differences between the language experience groups on infants’ habituation and test familiarization trial looking times, or the average number of habituation trials. However, bilingual infants (*M* = 13.33, SE = 2.00) looked significantly longer toward the baseline trial than did monolingual infants (*M* = 5.90, SE = 0.83), *t*(36) = 3.79, *p* = 0.001, Cohen’s *d* = 1.24.

The main question of interest was whether infants’ attention toward the target and distractor test trials in the different language condition differed depending on whether infants regularly received monolingual or bilingual linguistic experience. To investigate this question, we ran a 2 (test trial type: target, distractor) × 2 (linguistic experience: monolingual, bilingual) × 2 (first test trial: target, distractor) mixed-design ANOVA on infants’ attention toward the test trials with test trial type as the within-subjects factor. The results revealed a significant two-way interaction between linguistic experience and test trial type, *F*(1,34) = 6.38, *p* = 0.02, η^2^ = 0.16 (see Figure 2), and no other significant effects. Infants who are regularly exposed to more than one language looked significantly longer toward the target test trials than they did toward the distractor test trials, whereas infants who are only regularly exposed to one language did not look significantly longer toward either type of test trial.^4^

A chi-square analysis on the number of infants in each linguistic group who demonstrated longer looking toward (i.e., a preference for) the target test trials revealed that 81% of the infants being raised in multilingual environments, but only 41% of the infants being raised in monolingual environments, showed a looking time preference toward the target test trials, Pearson Chi-Square = 6.18, df = 1, *p* = 0.01. These results further confirm that experience influences infants’ expectations regarding the extent to which linguistic community constrains conventionality.

Lastly, a Pearson’s bivariate correlation revealed a significant negative correlation between the percentage of time infants are exposed to English and infants’ total looking time toward the target test trials, Pearson correlation = –0.34, *p* = 0.04. Consistent with the above results, infants who are exposed to English a lower percentage of time showed greater looking toward the target test trials.

The above set of analyses examined the role that varying levels of exposure to another language (i.e., from 0 to 60% exposure to a language other than English) plays on 13-month-old infants’ expectations of conventionality. The results demonstrate that linguistic experience influences infants’ expectation surrounding the extent to which linguistic community constrains conventionality. While infants being raised in monolingual environments do not generalize new words across speakers who use different languages, infants being raised in multilingual environments find it particularly surprising when speakers of different languages use the same word for the same object. These findings suggest that infants being exposed to more than one language might have an enhanced understanding of the fact that word meanings are generally unique to individual languages.

## Discussion

An understanding of the shared nature of word meanings emerges early in life (Woodward et al., 1994; Henderson and Graham, 2005; Graham et al., 2006; Buresh and Woodward, 2007; Henderson and Woodward, 2012). However, most of the evidence supporting this point comes from monolingual infants. As such, little is known about how linguistic experience influences this development. Given the growing number of families throughout the world that are raising their infants in multilingual environments, it is essential to understand how such experiences influence language development. The present research addresses this gap by examining whether exposure to more than one language influences infants’ developing expectations of conventionality. Specifically, we tested whether bilingual infants expect words to be shared across speakers who have been shown to use the same, or a different, language. If bilingual infants’ understanding of conventionality is similar to monolingual infants, we expected that bilingual 13-month-olds would assume that word-object associations would be consistent across users of the same language, but not users of different languages. To the best of our knowledge, our findings provide the first evidence that experience in bilingual environments influences the expectations that are formed about the shared nature of word meanings within the first 13 months of our lives.

Firstly, and contrary to our expectations, our findings suggest that bilingual infants do not have a robust expectation that word-object links should be consistent across speakers of the same language. This comes from our finding that infants in the same language condition did not look reliably longer toward either type of test trial and thus, demonstrating that they do not expect two speakers who use the same language to provide the same word-object pairings. This result contrasts with the results of previous research in which monolingual infants in the same condition look longer toward distractor test trials thereby demonstrating an expectation that word-referent pairings should be consistent across speakers who have been shown to use the same language (Henderson and Graham, 2005; Graham et al., 2006; Buresh and Woodward, 2007; Henderson and Woodward, 2012). This finding also appears to contrast with the findings reported by Byers-Heinlein et al. (2014) who posited that bilingual 2-year-olds’ tendency to avoid attaching a second label to an already labeled object revealed an assumption of conventionality. However, because the test trials in their study were completed by the same speaker and not a second speaker who spoke the same language as the first speaker, the extent to which the bilingual toddlers were performing in line with an assumption of conventionality remains unclear.

The finding that bilingual infants in the present study did not generalize word-object pairings across speakers of the same language suggests that exposure to more than one language encourages infants to be more conservative in assuming conventionality. Why might exposure to more than one language influence infants’ expectations of conventionality regarding two people who use the same language? One possibility is that bilingual infants might assume that other language users are also exposed to multiple languages, even though infants are only shown the speakers speaking one language. As such, bilingual infants might be more hesitant to generalize word meanings across speakers. This possibility is consistent with previous research in which bilingual preschool-aged children were less likely to assume conventionality and more likely to accept second labels for the same object than were the monolingual children (e.g., Diesendruck, 2005; Kalashnikova et al., 2014). Further, Pitts et al. (2015) revealed that 20-month-old monolingual infants assumed that unfamiliar people would only understand one language, whereas bilingual infants did not. Pitts et al. (2015) argued that their findings suggest that bilingual infants are more open to the possibility that an unfamiliar person could understand more than one language than are their same-aged monolingual peers. Taken together, our findings and those reported in previous research suggest that early consistent exposure to more than one language influences the extent to which children will assume conventionality.

Our finding that bilingual infants do not have the same expectations of conventionality as their same-aged monolingual peers aligns with research demonstrating that bilingual and monolingual infants have different expectations about the possible meanings of new words. For example, by 18 months monolingual infants demonstrate a robust expectation that novel words map on to novel objects (Halberda, 2003, 2006; Markman et al., 2003; Byers-Heinlein and Werker, 2009; Xu et al., 2011), whereas infants of the same age who are regularly exposed to more than one language do not (Byers-Heinlein and Werker, 2009; Houston-Price et al., 2010). Together with the present findings, existing evidence suggests that exposure to more than one language affects infants’ developing expectations about the meanings of words in several ways. In our future work we will seek to clarify the nature of the relationship between multilingual infants’ developing expectations about word meanings and the speakers who share them.

The second key finding from the present study is that bilingual infants do not expect two speakers who had been shown to speak different languages to use the same word to refer to the same object. Consistent with the findings reported by Scott and Henderson (2013) our results further confirm that 13-month-old infants appreciate that linguistic community constrains conventionality. However, our findings extend this past work by demonstrating that infants who are regularly exposed to more than one language are particularly sensitive to the constraints that the language an individual speaks constrains conventionality. This conclusion is supported by the fact that bilingual infants in the present study looked significantly longer toward the target test trials suggesting that they were particularly surprised when the two speakers who had been shown to use different languages knew (and used) the same word. This pattern contrasts with the pattern demonstrated by the monolingual infants in Scott and Henderson’s study who did not look reliably longer toward either type of test trial.

Longer looking toward the target test trials as a function of language experience was further confirmed in our final set of analyses directly comparing monolingual and bilingual infants. Infants who were exposed to at least one other language a greater percentage of time were more likely to look longer when users of different languages label objects consistently than were infants who were exposed only to English a greater percentage of time. The comparison of bilingual infants in the different language condition with a group of monolingual infants in the same condition provides converging evidence that exposure to more than one language enhances infants’ expectation that word meanings are tied to particular languages. Bilingual infants’ enhanced sensitivity to the fact that users of different languages do not share word-referent links is consistent with the recent findings reported by Byers-Heinlein et al. (2014) who showed that bilingual 2-year-olds have an enhanced understanding of the nature of foreign language words, compared to their same aged-monolingual peers, in a mutual exclusivity paradigm.

The unexpected finding that the bilingual infants in the different language condition looked longer toward the baseline trial than did the bilingual infants in the same language condition and the monolingual infants in the different language condition warrants some attention. This finding suggests that providing bilingual infants with a context in which they had been shown two speakers using two different languages heightened their attention toward the labeling event, but only during the baseline trial. One possibility is that the bilingual infants in the different language condition needed extra time after habituating to make sure that the speaker was labeling the object consistently to ensure that they could learn the new label for the object^5^. This finding was surprising and the reason for this difference is unclear. However, it is important to note that none of the other pre-test trial measures revealed significant differences between conditions and perhaps most importantly, the key difference between the two bilingual conditions in infants’ looking times toward the different test trials held after controlling for infants’ attention toward the baseline trial.

These findings raise interesting questions about which aspects of bilingual infants’ linguistic experience contribute to their enhanced understanding that word-referent links are not shared across different languages. One possibility is that bilingual infants’ own communicative experiences, perhaps of producing words in one language to speakers of a different language and possibly being misunderstood, play a key role in shaping their reduced tendency assume conventionality. However, the fact that infants in the present study were only 13 months of age and thus, would not have had significant amounts of experience producing their own words, suggests that simply being exposed to more than one language on a regular basis might be sufficient to raise questions about conventionality in bilingual infants. If this were true, then younger infants being raised in bilingual environments might also have different expectations about conventionality than their same-aged monolingual peers who have been shown to expect words to be shared across speakers of the same language (Henderson and Woodward, 2012). Indeed, future work investigating the developmental trajectory of this understanding will shed important insights into the kinds of experiences that support infants’ developing expectations about conventionality.

Another open question is whether the bilingual infants in this study would have performed differently if they were familiarized to two speakers who spoke both of the languages to which they are regularly exposed. In the present study all infants in the different language condition were exposed to an English speaker and a French speaker. As such most of the infants in the present research were only familiar with one of the languages used in the study (i.e., English). One interesting question is whether infants would have generalized the word-referent link if the other speaker had been shown to speak a language consistent with the other language to which infants were exposed. However, evidence from past research gives us reason to suspect that testing this possibility would not result in a different pattern of results. For example, in Scott and Henderson (2013), monolingual infants did not generalize word-referent links across users of different languages even when the English speaker completed the habituation phase. Thus, learning a new word-object pairing from a speaker from the same linguistic group as the infant did not result in the monolingual infants being more likely generalize the word-referent link across speakers who use different languages. Further, in their study on bilingual infants’ expectations of the communicative nature of foreign languages, Pitts et al. (2015) directly tested whether infants’ performance differed depending on their specific language-learning combinations. Their results revealed that bilingual infants’ performance did not differ when the test languages used in the study were more, or less, similar to infants’ own language-learning combination. Given these findings, we think it unlikely that our results would have been different if our participants had been familiar to both languages.

Our findings raise interesting questions about the extent to which bilingual infants’ expectations about conventionality influences their subsequent word learning. One way in which an understanding of conventionality has been argued to help children’s word learning is by enabling them to rapidly generalize words across both individuals and contexts (Sabbagh and Henderson, 2007, 2013). If bilingual infants do not expect words to generalize across individuals who use the same language, this might mean that they would require explicit information about a speaker’s awareness of particular word-referent links, a requirement that might result in slower word learning relative to their monolingual same-aged peers. However, the strategy of not assuming conventionality might also be adaptive as it might help bilingual word learners to keep track of specific word-referent links and the language to which they belong. Such a possibility is related to a second way in which an understanding of conventionality might be important for word learning; it might help children focus on learning the new word-referent links that will be relevant (i.e., shared) within the their own linguistic community (Sabbagh and Henderson, 2007, 2013). Regarding this point, our finding that bilingual infants might be more attuned to the fact that people who use different languages should not produce the same word-referent links, might mean that they might be better able to identify words that are unlikely to be shared by other language users. In so doing, they might be better able to streamline their word learning toward learning the word meanings that are most likely to be relevant within one of their linguistic groups. The extent to which an understanding of conventionality influences children’s word learning remains an open question. Future work examining the links between the development of an understanding of conventionality and its ties to word learning in children from both monolingual and bilingual backgrounds would help tease apart these potential consequences of multilingual exposure. Such work would determine whether conservatism when it comes to assuming conventionality is adaptive, or hinders subsequent language development.

In order for words to be effective communicative tools, their meanings must be known and used by all members within a given linguistic community. Adults readily appreciate this fact about language as we construct sentences using conventionally appropriate meanings and understand that communication will likely be difficult when speaking with people from other linguistic groups. There is now a substantial body of evidence suggesting that monolingual infants are sensitive to the conventional nature of language early in their lives. However, little is known about how diverse linguistic experiences influence infants’ understanding of conventionality. The present research provides the first evidence that linguistic experience influences the assumptions that children develop about the conventional nature of word meanings within the first year of their lives. Exposure to more than one language encourages infants to be more restrictive in their assumptions regarding conventionality within users of the same language and enhances their understanding that different languages constrain conventionality. This increased sensitivity to the constraints of conventionality represents a fairly sophisticated understanding of language as a conventional system and may play a role in shaping bilingual infants’ language development in a number of important ways.

### Conflict of Interest Statement

The authors declare that the research was conducted in the absence of any commercial or financial relationships that could be construed as a potential conflict of interest.

## Footnotes

1 The diversity in languages to which infants in this sample were exposed was a result of our participant recruitment approach. Many of these infants were called in to be included in the sample described in Scott and Henderson (2013). However, parents’ responses on our demographic questionnaire revealed that these infants did not meet the monolingual criteria.

2 These analyses were collapsed across gender, target object, and test pair as preliminary analyses revealed no significant interactions between these factors and condition or test trial type.

3 Scott and Henderson (2013) used the same videos as per the different language condition in the present study with infants who were primarily exposed to English (English exposure (> 80%).

4 An ANCOVA with attention toward baseline as the covariate, revealed a similar pattern however, the 2-way interaction between linguistic experience and test trial type was only approaching significance (*p* = 0.09).

5 We thank a reviewer for offering this suggestion.
